# Unforgiveness Motivations in Romantic Relationships Experiencing Infidelity: Negative Affect and Anxious Attachment to the Partner as Predictors

**DOI:** 10.3389/fpsyg.2019.00434

**Published:** 2019-03-22

**Authors:** Ana M. Beltrán-Morillas, Inmaculada Valor-Segura, Francisca Expósito

**Affiliations:** Mind, Brain and Behavior Research Center, Department of Social Psychology, University of Granada, Granada, Spain

**Keywords:** anxious attachment, infidelity, negative affect, romantic relationships, unforgiveness

## Abstract

Infidelity is considered an unforgivable betrayal. However, not all behaviors considered unfaithful affect the person who suffers them in the same way. Therefore, to have a better understanding of unforgiveness according to different extradyadic behaviors, two studies were designed. Study 1 (*N* = 240) explored which extradyadic behaviors are considered as more indicative of infidelity. The results revealed that sexual behaviors were considered more unfaithful when compared with technological, emotional/affective, and solitary behaviors. Study 2 (*N* = 378) examined the influence of experienced extradyadic behaviors on unforgiveness, negative affect, and anxious attachment to the partner. The results showed that (a) sexual and technological behaviors were less frequently forgiven and promoted a more intense negative affect, (b) anxious attachment was predictive of unforgiveness for sexual and technological behaviors, and (c) negative affect mediated the relationship between anxious attachment and unforgiveness for sexual and technological behaviors. These findings and their possible implications for romantic relationships are discussed.

## Introduction

Of the many betrayals that can occur within the context of romantic relationships, infidelity is considered the most severe and threatening to the stability of the relationship (Dillow et al., 2011; Beltrán-Morillas et al., 2015). This is typically considered an act of unforgivable betrayal, given the high expectations of loyalty and commitment that people hold for their partners (Watkins and Boon, 2016; Fincham and May, 2017) and the time and effort invested in preserving their relationships (Dillow et al., 2011; Fife et al., 2013). In other words, people usually acquire a relational commitment with their partners (i.e., they share property, experiences, time, children, etc.), so they do not expect their partners to engage in acts of intolerable betrayal, such as infidelity (e.g., Dillow et al., 2011; Watkins and Boon, 2016). However, not all types of infidelity are likely to affect people in the same way, given the wide variety of extradyadic behaviors that can be considered unfaithful (Thompson and O’Sullivan, 2016a). Moreover, within the context of romantic relationships, variables such as anxious attachment to the partner and negative affect have been positively related to unforgiveness (e.g., Prieto-Ursúa et al., 2012; Kimmes and Durtschi, 2016). Nevertheless, although these data shed light on how anxious attachment and negative affect are associated with non-absolution, the way in which these variables are related to unforgiveness motivations (revenge and avoidance) when people are faced with various extradyadic behaviors has not yet been addressed. Therefore, the main objective of this research is to examine the role of extradyadic behaviors as well as the anxious attachment and negative affect of the offended person on unforgiveness motivations toward the transgressive partner.

## Betrayal of Infidelity: Judgments and Associated Behaviors

Although there are many definitions that have been proposed to explain the concept of infidelity, there is still no agreement regarding its meaning (e.g., Thompson and O’Sullivan, 2016b; Thompson et al., 2017). An acceptable definition of infidelity could refer to it as a violation of the commitment of relational exclusivity, which can adopt a sexual, emotional, and/or mixed format of short or long duration (Dillow et al., 2011; Fife et al., 2013), with people other than the main partner (Dillow et al., 2011). However, one aspect to be taken into consideration is that the concept of infidelity could differ depending on the culture (e.g., whereas infidelity is not accepted in Western countries such as Spain, Britain, or the United States, Eastern countries such as Thailand tend to be more tolerant because sex has traditionally been commercialized and acceptance of extradyadic sex has increased; Mackay, 2001) and the type of relationship established (e.g., polyamorous couples or some same-sex couples who conceive their relationships away from the traditional romantic relationships and create explicit marriage agreements to make compulsory extradyadic behaviors; Martell and Prince, 2005; Moller and Vossler, 2015). Similarly, this definition is not acceptable if a diversity of opinions and judgments about behaviors that can be considered unfaithful are considered, because they are usually met with some disagreement from one person to another depending on his or her involvement or not with episodes of infidelity. Thus, people tend to judge their partner’s behavior as more indicative of infidelity than their own behavior (Thompson and O’Sullivan, 2016b). Thompson and O’Sullivan (2016a) classified different extradyadic behaviors that people believe to be constitutive of infidelity, establishing four groups of behaviors: (a) behaviors of a sexual nature (e.g., vaginal and or anal penetration or oral sex); (b) technological (e.g., sending someone sexually explicit and or affectionate text messages or emails); (c) emotional/affectionate (e.g., sharing secrets with a person other than the partner); and (d) solitary (e.g., masturbation). Under this classification, recent research shows that behaviors of a sexual nature are judged to be more indicative of infidelity because they tend to include more explicit behaviors and are not ambiguous (Thompson and O’Sullivan, 2016b, 2017; Thompson et al., 2017). Instead, evaluations based on patterns involving technological, emotional/affectionate, and solitary behaviors are usually considered more ambiguous behaviors and judged as indicative of infidelity depending on the particularities of the situation which occurred (Thompson and O’Sullivan, 2016b, 2017).

According to what was mentioned in the previous paragraph, infidelity of a sexual nature is seen as the most serious and the least likely to be forgiven (Pettijohn and Ndoni, 2013; Beltrán-Morillas et al., 2015). However, with the development of new technologies, the way in which people communicate and access information has changed, which has a considerable impact on romantic relationships (Clayton, 2014). That is, the opportunities to get involved in a relationship other than the primary relationship have increased, as extradyadic behaviors that make up this type of infidelity are easier to cover and deny (Vossler, 2016). Therefore, although infidelity has traditionally been approached from a perspective that implies purely sexual and emotional behaviors, new forms of infidelity are currently being incorporated that involve adulterous behavior of a technological nature, and they also have very harmful consequences for the person who suffers them (Henline et al., 2007; Vossler, 2016). In this context, one of the most agreed-upon definitions of technological infidelity was suggested by Hertlein and Piercy (2008), who defined it as “a romantic or sexual contact facilitated by Internet use that is seen by at least one partner as an unacceptable breach of their marital contract of faithfulness” (p. 484). In this regard, several authors have claimed that a nuance that has remained unchanged in the different definitions is the secret (e.g., people who perform extradyadic behavior can remove applications from their smartphones without leaving clear evidence of their existence because they do not show activity history; Hertlein and Piercy, 2006; Schneider et al., 2012).

A recent theoretical–empirical review by Vossler (2016) about the impact of technological infidelity revealed that betraying or deceiving the partner through this type of behavior has devastating effects, in some situations more significant than traditional sexual infidelity (Zitzman and Butler, 2005; Schneider et al., 2012). In this way, people who suffer technological infidelity tend to consider it a real episode of infidelity (Whitty and Quigley, 2008), which raises in the offended person the imperative need to demand therapeutic assistance to face the resulting traumatic impact (Schneider et al., 2012). This impact could be considered from the family ecological perspective, which focuses on the environmental result of ecological influences in romantic and family relationships. More specifically, the family ecological perspective emphasizes how the use of the Internet and new technologies generates changes in the way members of the couple or the family relate (Hertlein and Stevenson, 2010; Hertlein and Blumer, 2014). Thus, Hertlein and Stevenson (2010) conducted an in-depth review of the factors that represent the individual ecological vulnerabilities derived from technological infidelity and revealed the existence of seven factors known as the “Seven As”: anonymity (i.e., people can hide their true identity), accessibility (i.e., people have access to social networks and the Internet from different areas, and can interact with other people), affordability (i.e., Internet products and applications can be downloaded at a very low cost), approximation (i.e., social networks and the Internet let people meet each other face-to-face outside the virtual world), acceptability (i.e., people can develop romantic relationships through new technologies because they are usually a means of common use), accommodation (i.e., new technologies provide people with new opportunities to behave according to their true self, rather than as they should be), and ambiguity (i.e., communication and determining some behaviors as problematic or questionable may vary between people). Such factors have shown severe consequences for people who suffer this type of extradyadic behavior (Hertlein and Blumer, 2014). Consequently, technological infidelity—like traditional sexual infidelity—induces strong negative feelings in the offended person (e.g., feelings of anger, fear, shame or guilt; Whitty, 2005; Zitzman and Butler, 2005; Schneider et al., 2012), undermines marital quality, and results in loss of trust in the partner (e.g., Whitty, 2005; Schneider et al., 2012; Valenzuela et al., 2014), commonly concluding in separation or divorce (Whitty, 2005). In this regard, for instance, Whitty (2005) analyzed the perceptions of technological infidelity and its impact on the romantic relationship and found that participants referred to technological behaviors as infidelity. Similarly, the results of the study indicated that the participants noticed similar effects to those reported for traditional sexual infidelity, such as guilt, shame, loss of trust in the partner, and ending the relationship.

At this point, it is not surprising that infidelity has been considered a common phenomenon that affects many couples regardless of their nature (e.g., marriage, cohabiting, or dating relationships; Treas and Giesen, 2000; Lishner et al., 2008; Fife et al., 2013), so much so that infidelity rates fluctuate significantly according to various studies (Baucom et al., 2006; Abrahamson et al., 2012; Watkins and Boon, 2016; Fincham and May, 2017), with estimations of its prevalence at just over 60% (Abrahamson et al., 2012; Thompson and O’Sullivan, 2016b). Around 40% is attributed to men and approximately 20–25% to women (Abrahamson et al., 2012; Thompson and O’Sullivan, 2016b; Fincham and May, 2017). As far as the Spanish population is concerned, a study conducted in 2015 by the Ipsos Institute of Research and Marketing revealed that 35% of men and 26% of women confessed to having been unfaithful to their partner at some point in their lives, resulting in a higher percentage than in other countries of the European Union. According to some data provided by the General Council of the Judiciary in 2016, the divorce rate in Spain has increased, alleging infidelity as one of the main reasons, together with the facilities to access the Internet and social networks as precursors of such extradyadic behaviors.

## Negative Affect and Anxious Attachment on Unforgiveness Motivations in the Face of Infidelity

Unforgiveness is a response that the offended person manifests as a result of an act of betrayal, transgression, or severe interpersonal offense which generates a stressful or threatening situation for the ego (e.g., Berry et al., 2005; Wenzel and Okimoto, 2010). According to Berry et al. (2005) unforgiveness covers different motivations oriented toward revenge and/or avoidance of the person who transgresses the personal limits. In this sense, the motivation for avoidance has been defined as “the attempt to reduce stress by regulating one’s emotions and cognitions about the situation (e.g., venting emotions, accepting the problem, reinterpretation, and rumination), which often means assigning a new meaning to the event” (Strelan and Wojtysiak, 2009, p. 99). On the other hand, motivation for revenge has been conceptualized in various ways. For example, Govier (2002) stated that “when we seek revenge, we seek satisfaction by attempting to harm the other (or associated persons) as a retaliatory measure” (p. 2). In the same way, other social psychologists define it as “the intention to see the transgressor suffer” (Schumann and Ross, 2010, p. 1193). Hence, according to various authors, the most common occurrence of revenge motivation is the willful intention to inflict damage on the person who transgresses (e.g., Frijda, 1994; Carlsmith et al., 2008; McCullough et al., 2013; Gausel et al., 2018). Considering the above motivations, Worthington and Scherer (2004) pointed out that when an interpersonal event ensues, the offended person experiences a sense of injustice that he or she tries to restore, either through evasive behavior or through a manifest motivation for revenge in the form of repressive or coercive behavior against the transgressor. However, such behaviors arise mainly when severe situations such as infidelity occur, which is perceived by the offended party as an unforgivable betrayal (Fitness, 2001; Morrissette, 2012).

In connection with the aforementioned issues, constructs such as negative affective state and anxious attachment to the partner could influence the type of initial motivation that the offended person manifests as a consequence of infidelity, as shown by numerous studies that examined the role of negative affect and anxious attachment in unforgiveness (e.g., Finkel et al., 2007; McCullough et al., 2007; Zhang et al., 2015; Kimmes and Durtschi, 2016). Negative affect tends to manifest itself naturally when one of the parties is hurt by the action of the other, which is called *ongoing negative affect* (ONA; Merolla, 2008). This negative emotional state induces in the offended person a subjective experience of “non-forgiveness” that leads him or her to respond with revenge or avoidance behaviors toward the other person (Prieto-Ursúa et al., 2012). Emotions such as anger, fear, guilt, or resentment have been related to a greater motivation for revenge (McCullough et al., 2007; Rijavec et al., 2013), whereas other emotions such as shame or sadness have been associated with greater motivation to avoid the offending person (Schmader and Lickel, 2006; Leventhal, 2008).

Conversely, the configuration of attachment relationships that is established in the early stages of life is considered a relevant particularity in human beings for the adequate development of affective and romantic bonds in adult life (Bowlby, 1973; Hazan and Shaver, 1987; Cirhinlioğlu et al., 2016). Thus, the style of attachment in adulthood may develop in a double slope: secure attachment (Hazan and Shaver, 1987; Cirhinlioğlu et al., 2016) or insecure attachment that, in turn, can result in an avoidant attachment style or an anxious attachment style (Mikulincer and Shaver, 2007; Cirhinlioğlu et al., 2016). More specifically, anxious attachment has been described as a deep yearning for intimacy, a high agitation about the feelings of the other person, and excessive fear of rejection or abandonment by the partner (Valor-Segura et al., 2009; Morey et al., 2013), conceiving the most pathological dimension of the concept of interpersonal dependency on the partner when related to higher incidence of suffering affective, depressive, and anxiety disorders (Valor-Segura et al., 2009). Empirical evidence has shown that people who are high in anxious attachment frequently exhibit different surveillance strategies (e.g., Mikulincer and Shaver, 2007; Simpson and Rholes, 2015), as well as try to behave in a way that attracts or brings them closer to their partner (e.g., getting involved in infidelity to get their partner’s attention; for further review see McDaniel et al., 2017). Focusing on the first aspect, it has been argued that people with high anxious attachment experience discomfort when their needs to approach the partner are not met, expressing hypervigilance, control and intrusion behaviors, which would be oriented toward achieving closeness, care and attention by the partner (Mikulincer and Shaver, 2007; Simpson and Rholes, 2015). However, with regard to the relationship between anxious attachment and unforgiveness, several studies have shown that people with high levels of anxious attachment are driven by strong motivations for avoidance and revenge toward the partner (Finkel et al., 2007; Kimmes and Durtschi, 2016), mainly when the situation is perceived as risky for the continuity of the relationship (e.g., witnessing the infidelity of the partner; see Besser and Priel, 2009). According to various scholars, this is related to the fact that these people have difficultly disassociating themselves from perceived threats to their relationship (Mikulincer et al., 2002). Consequently, people with high levels of anxious attachment seem to react against disturbing situations with intense negative emotions (Barry et al., 2007; Mikulincer and Shaver, 2007; Marshall et al., 2013) and a high motivation not to forgive his or her partner (Finkel et al., 2007; Besser and Priel, 2011) because he or she experiences higher levels of jealousy (Barry et al., 2007; Marshall et al., 2013).

More specifically, infidelity has been established as the main reason for divorce and conjugal violence (Fife et al., 2013; Watkins and Boon, 2016; Fincham and May, 2017), having a significant negative impact on both members of the relationship and especially on the person who suffers it (e.g., Fincham and May, 2017). Therefore, because infidelity is considered an act of serious and threatening betrayal for the continuity of the relationship—mainly sexual and technological, perceived as similar in terms of seriousness (e.g., Schneider et al., 2012; Vossler, 2016)—it is likely that the offended person with a high level of anxious attachment experiences an intense negative affect formed by various emotions, which, in turn, may be related to his or her motivation not to forgive his or her transgressive partner. It is worth noting that among the two types of insecure attachment, this research will focus on the construct of anxious attachment, because studies have indicated that anxious attachment is associated more with the predictive variables related to infidelity (e.g., Russell et al., 2013). In the same way, it is noteworthy to focus on the unforgiveness motivations, given that several studies have shown that unforgiveness is negatively associated with the psychological well-being of the offended person (e.g., Gordon et al., 2005; Kluwer and Karremans, 2009) because if he or she does not forgive his or her partner, then he or she sustains the debt established by the betrayal (Kluwer and Karremans, 2009).

## The Current Research

Research addressing the topic of infidelity is important; however, the vast majority of existing studies used a methodology of scenarios or forced choice dilemmas (e.g., Sabini and Green, 2004; Lishner et al., 2008; Pettijohn and Ndoni, 2013) and focused exclusively on sexual and emotional infidelity (e.g., Sabini and Green, 2004; Lishner et al., 2008; Pettijohn and Ndoni, 2013; Buss, 2018). Moreover, so far there is no evidence of researchers who have explored in the Spanish population the role of unforgiveness in relation to the various extradyadic behaviors experienced by the offended person. That is why, given the scarcity of studies that refer to infidelity in the Spanish context—despite infidelity being considered one of the main reasons for divorce—it is imperative to study the effects of this phenomenon on the Spanish population.

Most current studies regarding anxious attachment addressed the influence of this orientation on the performance of individual patterns of mate retention (e.g., Barbaro et al., 2016; McDaniel et al., 2017). Likewise, existing investigations on infidelity approached it from the perspective of the perpetration of this betrayal, that is, how anxious attachment can be a predictor of engaging in an act of infidelity (e.g., Russell et al., 2013; Drouin et al., 2015). However, to date there are no known studies that considered the relationship of anxious attachment to the partner and negative affect on the various motivations for unforgiveness (revenge and avoidance) based on the extradyadic behaviors that make up each type of infidelity and considering the perspective of the offended person. Therefore, to provide greater knowledge to this field of research, two studies were designed: The first study was a pilot study to explore what kind of behaviors the Spanish population judges as more indicative of infidelity. It was expected that sexual behaviors (vs. technological, emotional/affectionate, and solitary) would be evaluated as more constitutive of infidelity (Hypothesis 1).

The purpose of the second study was to examine the role of extradyadic behaviors, anxious attachment, and negative affect on unforgiveness (motivation for revenge and avoidance) toward the transgressive partner. Although sexual infidelity has been considered the most severe, and those behaviors are recognized more explicitly (e.g., Beltrán-Morillas et al., 2015; Thompson and O’Sullivan, 2016b, 2017), recent literature has shown that, when it comes to a real episode of infidelity, behaviors that involve patterns of a technological nature can cause similar or even greater affliction than sexual ones (e.g., Schneider et al., 2012; Vossler, 2016). Therefore, the study was intended to do the following: (a) analyze the role of the extradyadic behaviors suffered on the motivations for revenge and avoidance toward the partner, expecting to find greater unforgiveness in the face of behaviors that involve to sexual and technological infidelity (vs. emotional/affectionate and solitary; Hypothesis 2); (b) analyze the role of extradyadic behaviors experienced on negative affect, expecting to find more intense negative emotions in response to sexual and technological behaviors (vs. emotional/affectionate and solitary; Hypothesis 3); (c) analyze the role of extradyadic behaviors and anxious attachment on unforgiveness motivations (revenge and avoidance), expecting that anxious attachment would be predictive of less forgiveness of sexual and technological behaviors (vs. emotional/affectionate and solitary; Hypothesis 4); (d) analyze the role of extradyadic behaviors and negative affect on unforgiveness motivations, expecting that negative affect would be predictive of less forgiveness of sexual and technological behaviors (vs. emotional/affectionate and solitary; Hypothesis 5); and e) examine whether the relationship between anxious attachment and negative affect is associated, in turn, with greater unforgiveness, especially in the face of extradyadic behaviors of a sexual and technological nature (vs. emotional/affectionate and solitary; Hypothesis 6).

## Pilot Study (Study 1)

### Methods

#### Participants

The initial sample consisted of 240 participants from the general population (120 women and 120 men), aged between 18 and 58 years (*M* = 27.06, *SD* = 7.26). Eight participants were excluded from the analysis because they did not complete the measure of interest. Thus, the final sample consisted of 232 participants from the Spanish population who were currently in a relationship (118 women and 114 men), with an average age of 27.54 (*SD* = 7.72; range from 18 to 59). Of the sample, 59.5% reported maintaining a dating relationship, 31.9% were living with their partner, and 8.6% reported being in a marriage. The average duration of the relationship was 59.55 months (*SD* = 76.65). In addition, 31.9% reported having suffered an incident of infidelity at some point in their lives.

#### Design and Procedure

Participants voluntarily filled out an online questionnaire through the Qualtrics research platform and did received no monetary compensation for their participation. The research was disseminated through various platforms and social networks (Facebook and Twitter), requiring that participants were Spanish and were currently in a romantic relationship. Before they completed the questionnaire, they were informed that the general purpose of the study was to examine “different emotional and motivational aspects involved in maintaining interpersonal relationships.” They were also informed of the anonymity of their responses and were guaranteed total confidentiality. Then, to provide their consent, participants had to check a box with the statement, “*After being informed of the above, I agree to participate in the study*.” We would like to add the Participants gave informed consent in accordance with the Declaration of Helsinki. The research was carried out after receiving the approval of the Ethics Committee of the University of Granada.

An intra-subject factorial design was used involving an exploratory survey methodology (Balcells-Junyent, 1994) by means of which participants were asked to indicate the degree to which they believed that each of the different behaviors could be considered infidelity. The behaviors were grouped into four blocks (sexual, technological, emotional/affectionate, and solitary) according to the typology of extradyadic behavior proposed by Thompson and O’Sullivan (2016a).

#### Instruments

##### Sociodemographic characteristics

Data about sex, age, if they were currently in a relationship, the duration of the relationship, relationship status, and if they had ever experienced an incident of infidelity were collected.

##### The definitions of infidelity questionnaire (Thompson and O’Sullivan, 2016a)

This questionnaire consists of 32 items structured in four subscales describing the different extradyadic behaviors that can be considered unfaithful: sexual/explicit behavior (seven items; e.g., “Engaging in penile–vaginal intercourse with someone,” “Receiving oral sex from someone”); online/technological behaviors (seven items; e.g., “Sending sexually explicit messages by text or email to someone,” “Receiving affectionate/flirtatious texts or emails from someone”); emotional/affectionate behaviors (thirteen items; e.g., “Receiving close emotional support from someone,” “Sharing secrets with someone”); and solitary behaviors (five items; e.g., “Engaging in masturbation alone,” “Viewing pornographic magazines alone”). A translation and back-translation process was carried out (English–Spanish/Spanish–English) according to the usual standards. This measure has a Likert-type response format with seven response options ranging from 1 (*not at all unfaithful*) to 7 (*very unfaithful*). The original measure has demonstrated adequate psychometric properties, revealing an internal consistency of 0.95 for sexual behaviors, 0.91 for online/technological behaviors, 0.95 for emotional/affectionate behaviors, and.88 for solitary behaviors. It has also demonstrated test–retest reliability, with a 6-week interval = *r*(156) = 0.96, *p* < 0.001. The alpha coefficient obtained in the present study for the subscales was 0.94 for sexual behaviors, 0.91 for online/technological behaviors, 0.94 for emotional/affectionate behaviors, and 0.88 for solitary behaviors.

#### Analysis Strategy

To inquire about what kinds of behaviors are estimated to be more unfaithful, a repeated measures mixed-design analysis of variance (ANOVA) model was used, including as covariates the duration and status of the couple’s relationship as well as if they had suffered an incident of infidelity.

#### Results

##### Type of infidelity and extradyadic behaviors

To verify whether behaviors involving sexual patterns (vs. technological, emotional/affectionate, and solitary) are judged as more indicative of infidelity (Hypothesis 1), a repeated-measures mixed ANOVA was performed.^1^ In this analysis, the previously mentioned elements were included as covariates.

First, it should be noted that no significant results of sex were found, nor any interaction between the type of infidelity X sex on extradyadic behaviors. The results showed that the type of infidelity influenced the perception of extradyadic behaviors, *F*(1,227) = 342.28, *p* < 0.001, $\eta_{p}^{2}$ = 0.60, so that sexual behaviors were considered more constitutive of infidelity (*M* = 6.32, *SD* = 1.20), followed by technological (*M* = 5.23, *SD* = 1.51), emotional/affectionate (*M* = 1.54, *SD* = 0.77), and solitary behaviors (*M* = 1.35, *SD* = 0.73), confirming Hypothesis 1. Likewise, pairwise comparisons using the Bonferroni test revealed significant differences between all types of extradyadic behaviors, thus establishing distinctions between sexual/technological (*p* < 0.001, 95% CI [0.888,1.300]), sexual/emotional (*p* < 0.001, 95% CI [0.4.542,5.023]), sexual/solitary (*p* < 0.001, 95% CI [4.718,5.208]), technological/emotional (*p* < 0.001, 95% CI [3.443, 3.934]), technological/solitary (*p* < 0.001, 95% CI [3.602, 4.136]), and emotional/solitary behaviors (*p* < 0.001, 95% CI [0.062,0.298]). In addition, it is important to note that these results were found regardless of the relationship duration, relationship status, and experience with infidelity.

## Study 2

The results of the previous study show that sexual behaviors (vs. technological, emotional/affectionate, and solitary) are judged to be the most indicative of infidelity given their ostensible and severe character (Thompson and O’Sullivan, 2016b, 2017; Thompson et al., 2017). However, in light of the real experience of infidelity, extradyadic behaviors of a technological nature also have very harmful effects on the person who suffers them (Vossler, 2016), which encourages thinking about the harmful consequences that new technologies can have for romantic relationships. That is why, because both types of behaviors have notoriously negative consequences, a second study was designed to examine what kinds of variables are related to the fact that people experiencing certain extradyadic behaviors (sexual/technological vs. emotional/solitary) manifest motivations for revenge and avoidance, and therefore greater unforgiveness toward the transgressive partner.

### Methods

#### Participants

The initial sample consisted of 378 participants from the general population (206 women and 172 men), aged between 18 and 60 years (*M* = 28.11, *SD* = 7.09). As in Study 1, 28 participants were removed from the analyses because they did not complete the measures of interest. The final sample consisted of 350 participants from the Spanish population who were in a relationship at present (195 women and 155 men), with an average age of 28.93 years (*SD* = 7.35, range from 18 to 59) and an average relationship duration of 64.96 months (*SD* = 66.58). Of the participants, 51.4% indicated that they were engaged in a dating relationship, 32.9% were living with their partner, and 15.7% were married. Finally, the reported prevalence of unfaithful behaviors experienced by the participants was 53.4% for those of a sexual nature, 56.6% for technological, 98% for emotional/affectionate, and 98.3% for solitary behaviors.

#### Design and Procedure

The same procedure as in Study 1 was followed. The participants voluntarily filled out an online questionnaire through the Qualtrics research platform and did not receive monetary compensation for their participation. The research required that participants were Spanish and were currently in a romantic relationship and was disseminated through various platforms and social networks (Facebook and Twitter). Participants were informed that the general purpose of the study was to examine “different emotional and motivational aspects involved in maintaining interpersonal relationships.” They were also informed of the anonymity of their responses and were guaranteed total confidentiality. Then, to obtain their consent, participants had to check a box with the statement, “*After being informed of the above, I agree to participate in the study*.” As in Study 1, the participants gave informed consent in accordance with the Declaration of Helsinki. Similarly, the research was conducted after receiving the approval of the Ethics Committee of the University of Granada.

An intra-subject factorial design was used whereby participants were presented with the different types of extradyadic behaviors (Thompson and O’Sullivan, 2016a) and asked to indicate which of them they had ever experienced in their relationship. As in Study 1, such behaviors were grouped into blocks according to their nature (sexual, technological, emotional/affectionate, and solitary). First, participants responded to the measure of anxious attachment to the partner, and then, after each block of behaviors, participants answered the measures of negative affect and unforgiveness motivations. Participants who indicated not having experienced any of the extradyadic behaviors of a block in question passed directly to another block of behaviors.

#### Instruments

##### Sociodemographic characteristics

The same data as in Study 1 were collected.

##### Spouse-specific dependency scale (Rathus and O’Leary, 1997; Spanish version of Valor-Segura et al., 2009)

The anxious attachment subscale consisted of five items (e.g., “I feel rejected when my partner is very busy”). The response format is Likert-type with six response options ranging from 1 (*totally disagree*) to 6 (*totally agree*). This scale has demonstrated adequate psychometric properties in the measurement of the construct of anxious attachment to the partner in the Spanish context, showing an internal consistency of 0.90 in the adaptation of the scale to the Spanish population. For the sample, an alpha coefficient of 0.75 was obtained.

##### The definitions of infidelity questionnaire (Thompson and O’Sullivan, 2016a)

This questionnaire was described in Study 1. Unlike in Study 1, participants were asked to report which of the 32 extradyadic behaviors they had experienced in their relationship. Participants answered using a binary response format “No” and “Yes,” which were coded as 0 and 1, respectively. Then, a composite score was created with the number of extradyadic behaviors suffered. That is, the affirmative answers belonging to each category were added (sum of “Yes” responses). The alpha coefficients obtained in this study were 0.94 for sexual behaviors, 0.85 for technological behaviors, 0.82 for emotional/affectionate behaviors, and 0.61 for solitary behaviors. It is important to note that the internal reliability was calculated using the Kuder and Richardson Formula 20 (KR20), a particularity of the alpha coefficient used in special cases in which the items are binary measures (Kuder and Richardson, 1937).

##### The positive and negative affect schedule (Watson et al., 1988; Spanish adaptation of Sandín et al., 1999)

The Negative Affect subscale composed of 10 items assessing the negative affective state of the individual at a given time (e.g., “I felt sad,” “I felt anger”). It is a Likert-type response format with five options ranging from 1 (*nothing*) to 5 (*a lot*). For the present study, an alpha coefficient of 0.96 was obtained.

##### Transgression-related interpersonal motivations scale-12-item form (McCullough et al., 1998)

This scale assesses different motivations that people experience after an interpersonal offense. It consists of 12 items divided into two subscales: revenge (five items; e.g., “I will make him/her pay”) and avoidance (seven items; e.g., “I am finding it difficult to act warmly toward him/her”). The response format is Likert-type with five response options ranging from 1 (*totally disagree*) a 5 (*totally agree*). The scale has shown appropriate psychometric properties in studies developed with Spanish samples (e.g., Beltrán-Morillas et al., 2015). In this sample, an alpha coefficient of 0.91 was obtained for the revenge subscale and 0.96 for the avoidance subscale.

#### Analysis Strategy

**FIGURE 1:**
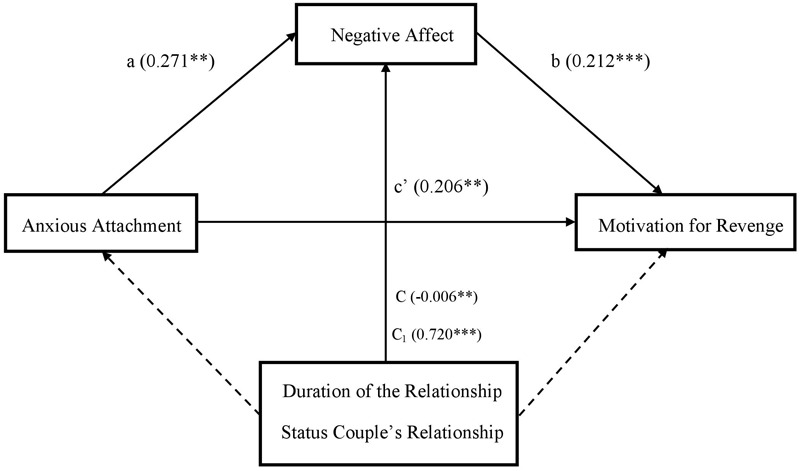
Study 2: Simple mediation model showing the direct effect of anxious attachment on motivation for revenge (c’), or the indirect effect through the mediator (a and b) on extradyadic behaviors of a sexual nature. The duration and status of the couple’s relationship, included as control variables (C and C_1_, respectively) affect only negative affect. ^∗^*p* < 0.05, ^∗∗^*p* < 0.01, ^∗∗∗^*p* < 0.001.

**FIGURE 2:**
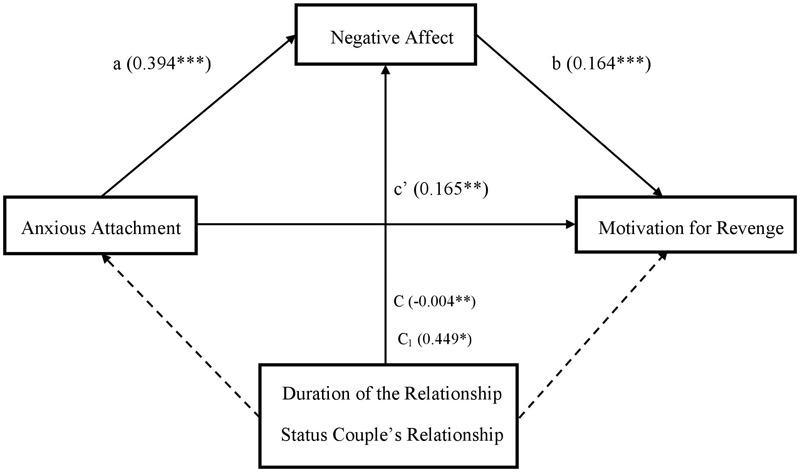
Study 2: Simple mediation model showing the direct effect of anxious attachment on motivation for revenge (c’), or the indirect effect through the mediator (a and b) on extradyadic behaviors of a technological nature. The duration and status of the couple’s relationship, included as control variables (C and C_1_, respectively) affect only negative affect. ^∗^*p* < 0.05, ^∗∗^*p* < 0.01, ^∗∗∗^*p* < 0.001.

First, to obtain information about the way in which the variables of interest are associated with each type of extradyadic behavior, different analyses of bivariate correlations were conducted (see Table 1). To inquire about what kind of extradyadic behaviors cause greater unforgiveness motivations, as well as a negative affective state of greater intensity, different repeated measures mixed-design ANOVAs were performed, including the duration and status of the couple’s relationship as covariates. Subsequently, to test the initial predictions about the role of anxious attachment and negative affect on unforgiveness motivations, mainly in sexual and technological extradyadic behaviors, a multiple linear regression analysis was implemented (see Tables 3, 4). Finally, to determine if the negative affective state mediates the relationship between anxious attachment and unforgiveness motivations in sexual and technological extradyadic behaviors (vs. emotional/affectionate and solitary), several simple mediation analyses were performed using Model 4 of the PROCESS macro program (Hayes, 2013; see Tables 5, 6 and Figure 1, 2). The duration and status of the couple’s relationship were included as covariates in said model.

**Table 1:** Descriptive statistics and correlations between the study variables for different extradyadic behaviors (Study 2).

	Sexual behaviors				Technological behaviors				Emotional behaviors				Solitary behaviors			
	1	2	3	4	1	2	3	4	1	2	3	4	1	2	3	4
(1) Motivation for Revenge	–				–				–				–			
(2) Motivation for Avoidance	0.58^∗∗^	–			0.52^∗∗^	–			0.62^∗∗^	–			0.58^∗∗^	–		
(3) Anxious Attachment	0.26^∗∗^	0.12	–		0.24^∗∗^	0.12	–		0.08	0.09	–		0.08	0.04	–	
(4) Negative Affect	0.43^∗∗^	0.54^∗∗^	0.20^∗∗^	–	0.36^∗∗^	0.46^∗∗^	0.30^∗∗^	–	0.41^∗∗^	0.50^∗∗^	0.33^∗∗^	–	0.29^∗∗^	0.40^∗∗^	0.18^∗∗^	–
*M*	1.54	2.79	2.41	3.04	1.40	2.46	2.41	2.76	1.15	1.49	2.41	1.59	1.13	1.34	2.41	1.43
*SD*	0.83	1.50	0.97	1.55	0.70	1.38	0.97	1.35	0.42	0.90	0.97	0.88	0.38	0.79	0.97	0.76

*^∗∗^*p* < 0.01.*

#### Results

##### Type of extradyadic behaviors and unforgiveness motivations (avoidance and revenge)

To examine whether the experienced behaviors of a sexual and technological nature (emotional/affectionate vs. solitary) give rise to greater unforgiveness motivations toward the partner (Hypothesis 2), a repeated-measures mixed ANOVA was carried out.^2^ In this analysis, the covariates referred to above were included.

In relation to sex, neither significant results nor results of interaction between the type of extradyadic behaviors and sex on avoidance and on revenge were found.

The results showed that the extradyadic behaviors influenced avoidance, *F*(1,127) = 7.97, *p* = 0.006, $\eta_{p}^{2}$ = 0.06; both sexual (*M* = 2.98, *SD* = 1.48) and technological behaviors (*M* = 2.55, *SD* = 1.42) encouraged a greater avoidance toward the transgressive partner, followed by emotional/affectionate (*M* = 1.70, *SD* = 1.09) and solitary behaviors (*M* = 1.52, *SD* = 1.00). Furthermore, through the Bonferroni test, pairwise comparisons revealed significant differences between all types of behaviors, except for emotional/affectionate and solitary behaviors (*p* > 0.05). Thus, differences between sexual/technological (*p* = 0.001, 95% CI [0.138,0.722]), sexual/emotional (*p* < 0.001, 95% CI [0.924,1.646]), sexual/solitary (*p* < 0.001, 95% CI [1.082,1.831]), technological/emotional (*p* < 0.001, 95% CI [0.499,1.211]), and technological/solitary behaviors (*p* < 0.001, 95% CI [0.704,1.349]) were obtained.

The results also showed that extradyadic behaviors influenced revenge, *F*(1,127) = 29.79, *p* < 0.001, $\eta_{p}^{2}$ = 0.19; sexual (*M* = 1.63, *SD* = 0.89) and technological unfaithful behaviors (*M* = 1.48, *SD* = 0.76) promoted a greater revenge toward the transgressive partner, followed by emotional/affectionate (*M* = 1.24, *SD* = 0.53) and solitary behaviors (*M* = 1.20, *SD* = 0.48). As with the previous result, pairwise comparisons showed significant differences among all types of behaviors, except for sexual and technological, and for emotional/affectionate and solitary behaviors (*p* > 0.05). In this way, differences were revealed between sexual/emotional (*p* < 0.001, 95% CI [0.222,0.574]), sexual/solitary (*p* < 0.001, 95% CI [0.233,0.645]), technological/emotional (*p* = 0.001, 95% CI [0.080,0.403]), and technological/solitary behaviors (*p* < 0.001, 95% CI [0.106,0.460]).

These results confirm Hypothesis 2. It is also noteworthy to point out that these results were obtained regardless of the duration and status of the couple’s relationship.

##### Type of extradyadic behaviors and negative affect

To analyze whether extradyadic behaviors of a sexual and technological nature (vs. emotional/affectionate and solitary) cause a more intense negative affect in the person experiencing them (Hypothesis 3), a repeated-measures mixed ANOVA was performed. The duration and status of the couple’s relationship were included as covariates.

The results revealed that extradyadic behaviors influenced negative affect, *F*(1,127) = 12.43, *p* = 0.001, $\eta_{p}^{2}$ = 0.09; behaviors of a sexual and technological nature (*M_sexual_* = 3.15, *SD* = 1.50; *M_technological_* = 2.81, *SD* = 1.46) provoked a more intense negative affect in the person who suffered them, followed by emotional/affectionate (*M* = 1.75, *SD* = 1.03) and solitary behaviors (*M* = 1.51, *SD* = 0.84). These findings verify Hypothesis 3. Likewise, it should be mentioned that these results were obtained regardless of the covariates identified above.

The results also showed an effect of interaction between the type of extradyadic behaviors and sex on negative affect, *F*(1,127) = 19.77, *p* < 0.001, $\eta_{p}^{2}$ = 0.13; women experienced a greater negative affect when faced with all types of extradyadic behaviors compared to men (see Table 2).

**Table 2:** Mean scores and standard deviations of the participants on negative affect according to the different types of extradyadic behaviors (Study 2).

		Women				Men			
		SB	TB	EB	SLB	SB	TB	EM	SLB
		*M(SD)*	*M(SD)*	*M(SD)*	*M(SD)*	*M(SD)*	*M(SD)*	*M(SD)*	*M(SD)*
Negative Affect		3.89 (0.16)	3.32 (0.17)	1.99 (0.13)	1.73 (0.10)	2.43 (0.16)	2.31 (0.17)	1.52 (0.12)	1.30 (0.10)

*SB, Sexual Behaviors; TB, Technological Behaviors; EB, Emotional Behaviors; SLB, Solitary Behaviors.*

The Bonferroni test showed significant differences between all types of extradyadic behavior, except for emotional/affectionate and solitary behaviors (*p* > 0.05). Thus, differences were found between sexual/technological (*p* = 0.002, 95% CI [0.095,0.593]), sexual/emotional (*p* < 0.001, 95% CI [1.132,1.680]), sexual/solitary (*p* < 0.001, 95% CI [1.341,1944]), technological/emotional (*p* < 0.001, 95% CI [.778, 1.346]), and technological/solitary behaviors (*p* < 0.001, 95% CI [0.993,1.605]).

##### Type of extradyadic behaviors, anxious attachment to the partner, and negative affect on unforgiveness motivations (avoidance and revenge)

To examine whether anxious attachment to the partner and negative affect are predictive of greater unforgiveness (motivation for avoidance and revenge), mainly in the face of sexual and technological behaviors (vs. emotional and solitary; Hypotheses 4 and 5), a multiple linear regression analysis was performed. The introduced predictive variables were sex (0 = men; 1 = women), anxious attachment and negative affect, and the motivations for revenge and avoidance were criteria variables. Similarly, the duration and status of the couple’s relationship were included as control variables. All scores were standardized before the corresponding analysis was performed, contrasting the effects of the control variables in the first step, the predictive variables in the second step, and interaction effects in relation to sex in the third step (see Tables 3, 4).

**Table 3:** Effect of anxious attachment and negative affect on motivation for avoidance depending on the type of extradyadic behaviors (Study 2).

Motivation for avoidance													
		SB			TB			EB			SLB		
		*β*	*t*	*p*	*β*	*t*	*p*	*β*	*t*	*p*	*β*	*T*	*p*
**Step 1**	Duration Relationship	-0.22	-2.30	0.023	-0.12	-1.40	0.163	-0.01	-0.03	0.974	0.00	0.06	0.949
	Status Couple’s Relationship	0.14	1.52	0.130	0.04	0.51	0.614	-0.04	-0.59	0.552	-0.02	-0.22	0.822
	*R^2^*	0.028			0.011			0.002			0.000		
	*Adjusted R^2^*	0.017			0.001			-0.004			-0.006		
**Step 2**	Sex	-0.15	2.16	0.032	-0.14	2.03	0.043	-0.04	-0.73	0.464	0.01	0.23	0.821
	Anxious Attachment	0.02	0.25	0.800	-0.01	-0.21	0.831	-0.08	-1.53	0.126	-0.04	-0.81	0.420
	Negative Affect	0.60	8.69	<0.001	0.52	7.25	<0.001	0.54	10.67	<0.001	0.43	8.34	<0.001
	*R^2^*	0.325			0.233			0.263			0.179		
	*Adjusted R^2^*	0.306			0.213			0.252			0.167		
**Step 3**	A. Attachment × Sex	0.15	1.60	0.111	-0.07	-0.69	0.492	-0.11	-1.46	0.144	-0.18	-2.40	0.117
	N. Affect × Sex	-0.02	-0.24	0.811	-0.05	-0.51	0.608	0.00	0.01	0.990	0.25	2.59	0.710
	*R^2^*	0.334			0.237			0.268			0.205		
	*Adjusted R^2^*	0.308			0.209			0.253			0.188		

*SB, Sexual Behaviors; TB, Technological Behaviors; EB, Emotional Behaviors; SLB, Solitary Behaviors.*

**Table 4:** Effect of anxious attachment and negative affect on motivation for revenge depending on the type of extradyadic behaviors (Study 2).

Motivation for revenge													
		**SB**			**TB**			**EM**			**SLB**		
		***β***	***t***	***p***	***β***	***t***	***p***	***β***	***t***	***p***	***β***	***t***	***p***

**Step 1**	Duration Relationship	-0.23	-2.44	0.016	-0.18	-2.08	0.039	0.04	0.56	0.573	0.04	0.56	0.575
	Status Couple’s Relationship	0.11	1.17	0.244	0.05	0.59	0.553	-0.06	-0.76	0.448	-0.07	-1.16	0.248
	*R^2^*	0.033			0.025			0.002			0.004		
	*Adjusted R^2^*	0.022			0.015			-0.004			-0.002		
**Step 2**	Sex	-0.11	-1.57	0.119	-0.07	-0.95	0.342	-0.09	-1.74	0.083	-0.08	-1.39	0.166
	Anxious Attachment	0.19	2.84	0.005	0.14	2.03	0.044	-0.05	-0.98	0.330	0.03	0.67	0.503
	Negative Affect	0.44	6.00	<0.001	0.34	4.60	<0.001	0.45	8.53	<0.001	0.31	5.70	<0.001
	*R^2^*	0.247			0.169			0.185			0.098		
	*Adjusted R^2^*	0.227			0.147			0.173			0.085		
**Step 3**	A. Attachment × Sex	0.12	1.22	0.223	0.13	1.26	0.207	0.02	0.28	0.781	-0.16	-2.02	0.344
	N. Affect × Sex	-0.09	-0.88	0.381	-0.02	-0.23	0.815	-0.04	-0.45	0.653	0.16	1.55	0.121
	*R^2^*	0.256			0.176			0.186			0.113		
	*Adjusted R^2^*	0.226			0.145			0.169			0.094		

*SB, Sexual Behaviors; TB, Technological Behaviors; EB, Emotional Behaviors; SLB, Solitary Behaviors.*

As seen in Table 3, in relation to “motivation for avoidance,” the results showed that negative affect is predictive of greater avoidance in all types of extradyadic behaviors: sexual (*β* = 0.60, *p* < 0.001), technological (*β* = 0.52, *p* < 0.001), emotional/affectionate (*β* = 0.54, *p* < 0.001), and solitary (*β* = 0.43, *p* < 0.001). People with intense negative affect seem to show higher motivation to avoid and, therefore, not to forgive their partner, which is indicative of infidelity. In addition, the results revealed a main effect of sex when faced with behaviors of a sexual (*β* = -0.15, *p* = 0.032) and technological nature (*β* = -0.14, *p* = 0.043); men (*M_SB_* = 2.91, *SD* = 1.62; *M_TB_* = 2.50, *SD* = 1.41) compared to women (*M_SB_* = 2.67, *SD* = 1.36; *M_TB_* = 2.42, *SD* = 1.34) seemed to exhibit greater motivation for avoidance and, therefore, not to forgive their partner in light of this kind of extradyadic behavior. The duration of the relationship, included as a covariate, was significant only for sexual behaviors (*β* = -0.22, *p* = 0.023). That is, a shorter time committed to the relationship predicts greater motivation to avoid the transgressive partner.

With respect to “motivation for revenge” (Table 4), the results revealed that anxious attachment is predictive of greater revenge in the face of behaviors of a sexual (*β* = 0.19, *p* = 0.005) and technological nature (*β* = 0.14, *p* = 0.044). People with high levels of anxious attachment seem to experience higher motivation to take revenge on their partner following sexual and technological extradyadic behaviors. Likewise, the results revealed that negative affect is predictive of greater revenge in all types of extradyadic behaviors: sexual (*β* = 0.44, *p* < 0.001), technological (*β* = 0.34, *p* < 0.001), emotional/affectionate (*β* = 0.45, *p* < 0.001), and solitary (*β* = 0.31, *p* < 0.001). People with high negative affect seem to have greater motivation to take revenge on their partner and, therefore, not to forgive him or her in light of different extradyadic behaviors. The duration of the relationship, introduced as a control variable, was significant for behaviors of a sexual (*β* = -0.23, *p* = 0.016) and technological nature (*β* = -0.18, *p* = 0.039); a shorter time committed to the relationship is predictive of greater motivation for revenge toward the transgressive partner.

These findings confirm Hypothesis 4 and partially support Hypothesis 5.

##### The mediating role of negative affect between anxious attachment and unforgiveness motivations (avoidance and revenge) on sexual and technological extradyadic behaviors

To examine Hypothesis 6, which predicted that negative affect would mediate the relationship between anxious attachment and motivation for revenge (unforgiveness), mainly in the face of extradyadic behaviors of a sexual and technological nature (vs. emotional/affectionate and solitary), Model 4 of the PROCESS macro program (Hayes, 2013) was used.^3^^,^^4^ This model enables testing the indirect effect of anxious attachment on motivation for revenge through negative affect. To this end, the recommendations of MacKinnon et al. (2004) were followed using the nonparametric bootstrapping procedure with 10,000 replicates to estimate the 95% confidence interval. The control variables included were the duration and status of the couple’s relationship.

The variables included in the model predicted 23.7 and 16.5% of the variance of the predisposition to show motivation for revenge against the partner in the face of sexual and technological behavior, respectively. As shown in Table 5, regarding motivation for revenge, the results of the mediation model obtained for sexual behaviors show that anxious attachment is positively related to negative affect and motivation for revenge, and negative affect is positively associated with revenge. Similarly, the results for technological extradyadic behaviors reveal that anxious attachment is positively related to negative affect, and negative affect is positively associated with revenge (Table 6). The 95% confidence interval based on the model of sexual behaviors was between 0.286 and 1.120, whereas the confidence interval on the model of technological behaviors was between 0.478 and 1.220. Therefore, according to initial predictions, the results showed that the indirect effect of anxious attachment on motivation for revenge through negative affect was significant, both for sexual and technological extradyadic behaviors (vs. emotional/affectionate and solitary), verifying Hypothesis 6. In both models, the duration of the relationship and status of the couple’s relationship were significant, corroborating the previous results mainly for people with shorter duration relationships and those living with their partner. The final models obtained are shown in Figure 1, 2.^5^

**Table 5:** Non-standardized regression coefficients, standard errors, and summary information for model 4 for simple mediation (Extradyadic Sexual Behaviors; Study 2).

	Negative affect				Motivation for revenge			
Background	Coeff.	*SE*	*t*	*p*	Coeff.	*SE*	*t*	*p*
Constant	1.563	0.39	3.98	<0.001	0.703	0.21	3.33	0.001
Anxious Attachment	0.271	0.10	2.60	0.010	0.149	0.06	2.32	0.021
Negative Affect					0.212	0.03	5.94	<0.001
Duration Relationship	-0.006	0.00	-3.18	0.002	-0.001	0.00	-1.05	0.295
Status Couple’s Relationship	0.720	0.20	3.51	<0.001	-0.054	0.10	-0.51	0.607
	*R^2^* = 0.105				*R^2^* = 0.237			
	*F*(3,183) = 7.26, *p* < 0.001				*F*(4,182) = 10.25, *p* < 0.001			

*SE, standard error.*

**Table 6:** Non-standardized regression coefficients, standard errors, and summary information for model 4 for simple mediation (Extradyadic Technological Behaviors; Study 2).

	Negative affect				Motivation for revenge			
Background	Coeff.	*SE*	*t*	*p*	Coeff.	*SE*	*t*	*p*
Constant	1.362	0.31	4.37	<0.001	0.849	0.19	4.51	<0.001
Anxious Attachment	0.394	0.09	4.23	<0.001	0.100	0.06	1.65	0.101
Negative Affect					0.164	0.03	4.58	<0.001
Duration Relationship	-0.004	0.00	-2.95	0.003	-0.001	0.00	-0.99	0.323
Status Couple’s Relationship	0.449	0.18	2.46	0.015	-0.054	0.08	-0.65	0.518
	*R^2^* = 0.127				*R^2^* = 0.165			
	*F*(3,195) = 11.18, *p* < 0.001				*F*(4,194) = 6.82, *p* < 0.001			

*SE, standard error.*

## Discussion

The present research explored, on the one hand, the types of extradyadic behaviors considered to be more constitutive of infidelity by the Spanish population and, on the other hand, variables influencing the unforgiveness motivations regarding extradyadic behaviors, such as negative affect and anxious attachment.

The results of Study 1 revealed that behaviors of a sexual nature are considered to be more constitutive of infidelity, followed by technological behaviors, which also received a high score compared to emotional/affectionate and solitary behaviors. These results are consistent with previous research showing that behaviors of a sexual nature are less ambiguous and more severe, which contributes to them being perceived as more indicative of infidelity (Mattingly et al., 2010; Wilson et al., 2011; Rodrigues et al., 2016; Thompson and O’Sullivan, 2016a,b). This perception is also supported by statistics, which reveal that between 70 and 90% of the population involved in both dating and marital relationships conceive sexual infidelity as an intolerable and intransigent betrayal and 65% perceive it as unforgivable (Whisman et al., 2007; Rodrigues et al., 2016; Thompson and O’Sullivan, 2016b). Regarding technological behaviors—and in spite of its ambiguous nature—the increased use of technology in recent years has significantly impacted couples’ lives, clarifying the perception of behaviors involving this kind of interpersonal communication (McDaniel and Coyne, 2014). Given the amount of technological means available to establish extradyadic relationships (e.g., access to Internet, social networks, or mobile phones; McDaniel and Coyne, 2014) and the ability to hide these relationships, the appreciation of certain technological behaviors that might promote an affair is increasing (e.g., sending to/receiving from another person affectionate/sexual messages; Henline et al., 2007; Schneider et al., 2012). It is interesting to point out that our results differ from those in the literature focused on ambiguous and deceptive behaviors. For example, previous studies revealed that ambiguous behaviors are considered less indicative of infidelity (e.g., Feldman and Cauffman, 1999; Wilson et al., 2011). However, such behaviors are constituted by both technological and emotional behaviors (e.g., talking on the phone or over the Internet, buying or receiving gifts). In our study, both categories were considered independently, evidencing that emotional behaviors are considered less indicative of infidelity compared to technological ones, probably due to the fact that emotional behaviors are perceived with greater ambiguity. Similarly, previous studies revealed that deceptive behaviors are considered moderately indicative of infidelity (e.g., Feldman and Cauffman, 1999; Wilson et al., 2011; Rodrigues et al., 2016). Such behaviors are intrapersonal processes (Mattingly et al., 2010) and could be considered in a certain way as solitary behaviors given that it is the individual who performs and receives the action. In this respect, our results differ from those of previous research, showing that solitary behaviors are considered less indicative of infidelity. However, it is possible that deceptive behaviors can also be accommodated in all categories because they involve denying or withholding information from the partner; this kind of information could be about any sexual, emotional, technological, or solitary behavior. Future research might inquire about this possibility.

An interesting finding of this study was that previous experience with infidelity does not affect the perception of extradyadic behavior as indicative of it. This could be due to the fact that the perception of extradyadic behavior could be influenced to a greater extent by social norms, because such behaviors tend to occur within a more collective and less situational context (e.g., infidelity may be less accepted at a social level; however, it is more tolerated within the relationship; Selterman and Koleva, 2015; Selterman et al., 2018). Future research could replicate these findings considering the role of social norms in the perception of extradyadic behaviors.

The results of Study 2 revealed firstly that, in light of the experimentation of different extradyadic behaviors, both those of a sexual and technological nature (vs. emotional/affectionate and solitary) promote a greater motivation for avoidance and revenge (unforgiveness) toward the transgressive partner. This finding could contribute to a better understanding of results found in previous research, showing that people who experience infidelity or extradyadic behavior of a technological nature consider this type of behavior equally or even more devastating and traumatizing than traditional sexual infidelity (e.g., Zitzman and Butler, 2005; Schneider et al., 2012). In this regard, Schneider et al. (2012) through a qualitative study with participants who had experienced technological extradyadic behavior through different media, analyzed the impact of this type of behavior on the offended person. The results revealed that, as in the case of traditional infidelity, the person suffering from such behaviors tends to lose trust in his or her partner, identify him or herself as a victim of a betrayal, and feel that he or she needs to seek help to overcome the pain caused by trauma. Accordingly, such effects could be related to an increase in unforgiveness motivations toward the transgressive partner. That is, given the magnitude of the severity of both types of behavior (sexual and technological), the offended person could find him or herself motivated to shy away from and disturb the partner or to react with greater revenge to restore the balance between his or her own suffering and that of the offending person (Frijda, 1994; Worthington and Scherer, 2004; Gausel et al., 2018). The results also revealed that men (vs. women) show a greater avoidance motivation in the face of sexual and technological behaviors. In this regard, Fincham et al. (2004) examined whether forgiveness was associated with improved conflict resolution in romantic relationships. Their results revealed that men scored higher in avoidance motivation, associating in turn with less face-to-face discussion, and a more elusive attitude toward conflicts. However, it is interesting that in light of a severe transgression such as infidelity, men use the motivation for avoidance to show unforgiveness to their partner. This finding could be affected by variables such as lack of commitment or the quality of alternatives outside of their primary relationship (Rusbult et al., 1998). In the same way, this motivation could be used as a form of rejection or contempt toward the partner (Cavallo et al., 2010; Bernecker et al., 2018). Further research is needed to address this finding.

With regard to negative affect, the results showed that sexual and technological extradyadic behaviors provoke a negative emotional state of greater intensity in the offended person. This finding could be related to previous research results showing that, as in the case of sexual infidelity, behaviors of technological infidelity are associated with a devastating emotional state promoted by emotions and feelings such as anger, humiliation, fear, sadness, guilt, shame, or rejection (Zitzman and Butler, 2009; Fincham and May, 2017), which, in turn, are related to states of confusion, excessive worry, loss of confidence in the romantic partner, or even sexual and depressive disorders (Schneider et al., 2012; Fincham and May, 2017). Similarly, the results showed an interaction between negative affect and sex, indicating that women (vs. men) scored higher in negative affect on all types of extradyadic behaviors. Women tend to have a greater relational orientation (Knox et al., 1997; Manning et al., 2006). This is why, faced with infidelity, they could react with greater negative emotions in face of the breach of trust by the partner, and increase their relational skills to end an undesirable relationship (Knox et al., 1997). This finding is novel given that a large number of studies showing sex effects in emotional reactions to infidelity did not consider negative affect and focused mainly on the level of distress and traditional types of infidelity (sexual vs. emotional; e.g., Wade et al., 2012; Tagler, 2013). Likewise, the results revealed that negative affect is predictive both of a greater motivation for revenge and of avoidance in all types of extradyadic behaviors. This finding is not exceptional if one takes into account the variety of behaviors judged to be unfaithful and if one considers the magnitude of the aversive emotional impact resulting from infidelity (e.g., Thompson and O’Sullivan, 2016a; Fincham and May, 2017). Thus, the offended person motivated by unforgiveness could be tempted to respond with greater resentment, retaliation, and/or avoidance toward the partner (Kluwer and Karremans, 2009).

In relation to anxious attachment to the partner, the results showed that it is predictive of a greater motivation for revenge in light of extradyadic behaviors of a sexual and technological nature. A possible explanation could be that this type of people tends to be excessively preoccupied with possible rejection and/or abandonment, so they tend to increase the level of monitoring and control if they feel their partner is not receptive (Barry et al., 2007; Marshall et al., 2013). Consequently, given the suspicion that their partner can maintain a parallel relationship (Guerrero, 1998), they could respond with greater vengeful behavior (Besser and Priel, 2011), which could be oriented toward restoring the sense of injustice and mitigating the discomfort caused by the situation of infidelity (Fitness, 2001; Morrissette, 2012).

Lastly, the results showed that when faced with extradyadic sexual and technological behaviors (vs. emotional/affectionate and solitary), anxious attachment is related to a greater negative affect that, in turn, is associated with a greater motivation for revenge toward the transgressive partner. People with a high level of anxious attachment tend to be more distrustful of their partner and live constantly afraid that they will be abandoned or rejected by a third party (i.e., they may feel rejected or abandoned if their partner leaves them for someone else; Marshall et al., 2013). Hence, people high in anxious attachment could increase their supervision of extradyadic threats and warn of sexual infidelity—considered the most severe relational transgression (Beltrán-Morillas et al., 2015)—as a danger to the continuity of the relationship. Given this situation, these people might experience intense negative affect and react with more aggressive behaviors motivated by the motivation for revenge or unforgiveness toward the partner (Wang et al., 2012; Kimmes and Durtschi, 2016). In the case of technological infidelity, this finding is even more interesting and highlights the relevance of using new technologies in the romantic relational context. People with high anxious attachment, given that they exhibit excessive concern about the state of their relationship, are likely to make use of different ways to be in constant contact with their partner to ensure relational fidelity (e.g., social networks or smartphones; Morey et al., 2013), which could lead them to experience intense negative emotions and to react accordingly with a pronounced motivation for revenge faced with the suspicion of a third person (Besser and Priel, 2011; Marshall et al., 2013). These results are also affected by the duration (low duration) and status of the couple’s relationship (living together). On one hand, according to the investment model (Rusbult et al., 1998), the duration of the relationship is directly related to the level of commitment, and the amount of resources invested in the relationship. Therefore, at the beginning of the relationship there is usually little commitment and investment, as well as high uncertainty, with jealousy manifesting itself as a way of preserving the relationship (Rusbult, 1983). Similarly, it has also been shown that the lower the level of commitment, the lower the probability of forgiving the transgressive partner (e.g., Braithwaite et al., 2011). On the other hand, the average duration of the relationship in this study was just over 5 years; thus, it is possible that couples living together were still in the early stages of their romantic relationship and had not yet developed commitment and/or attachment (e.g., Fincham et al., 2007). Therefore, in this study it could be feasible that the duration of the relationship is affected by the level of commitment and relational investment, which could influence both the negative affect and unforgiveness motivations of the offended person suffering infidelity. Future research could consider these variables, as well as others such as the expectations regarding the relationship, or the level of trust in the partner (Luchies et al., 2013; Lemay and Venaglia, 2016).

An unexpected finding in Study 2 was that mediation was not significant for avoidance motivation. Some research suggests that the stimulus that prevails in people suffering infidelity is retaliation or revenge against the partner because, in this way, the offended person fights the pain he or she experiences as a result of the betrayal (Fitness, 2001; Morrissette, 2012). Such a response could be appreciable in people with high anxious attachment as a result of the fear they show of their partner rejecting or abandoning them for a third person (Marshall et al., 2013). Meanwhile, motivation for avoidance would be more oriented toward acceptance and reinterpretation of the situation that occurred in order to give new meaning to the event (Strelan and Wojtysiak, 2009). This could be related to a greater extent with one of the stages of the infidelity healing process (Fife et al., 2013). However, more research is needed in this area to clarify the role of motivation for avoidance in people with anxious attachment who have experienced infidelity, as well as to examine if this motivation could be a step in the process of healing from the infidelity.

## Limitations and Future Directions

Although this work complements existing findings and contributes to improved understanding of unforgiveness in light of infidelity, it is not exempt from limitations, which will attempt to be resolved in future research. Despite being a non-experimental study, the data obtained were correlational and, therefore, could not indicate causal relationships or be generalized to the total population. Future studies could replicate these findings to determine whether they can be generalized beyond the Spanish context. Future studies could also test these results by intentional sampling to select and compare different groups (e.g., dating and marital relationships; Slater, 2013; Turkle, 2015) because, despite the fact that the use of technologies is becoming more frequent, the debate about the effect of technology-related behaviors has been hampered by a lack of representative data for primary relationships (Rosenfeld, 2017).

In addition, future studies might consider other variables to help understand the present findings and could be substantial for both relationship processes. For instance, research has shown that people who have an unrestrained sexual orientation perceive certain extradyadic behaviors as less indicative of infidelity (Mattingly et al., 2010; Rodrigues et al., 2016), have a greater predisposition to engage in extradyadic behaviors (Rodrigues et al., 2016; Weiser et al., 2018), and accept to a greater extent the infidelity of the partner (Sharpe et al., 2013). For its part, the level of commitment can also influence the perception of extradyadic behaviors as indicative of infidelity. In this sense, studies have revealed that a high level of commitment is related to more restrictive behavior toward infidelity, and greater perception of extradyadic sexual behaviors as indicative of it (e.g., Rodrigues et al., 2016). The results of these investigations are mainly focused on sexual and technological behaviors (e.g., Mattingly et al., 2010; Rodrigues et al., 2016; Weiser et al., 2018), so future studies could replicate our findings considering the variables above, as well as including emotional and solitary behaviors.

Similarly, several studies showed that the higher the level of commitment, the greater the likelihood that the offended person forgives his or her partner after a transgression (e.g., McCullough et al., 1998; Braithwaite et al., 2011). However, the path by which both variables are related seems to be inconclusive. Empirical evidence has revealed that the level of commitment may be affected by the degree of shock that people experience after infidelity (Marcussen et al., 2004; Heintzelman et al., 2014), thus affecting their levels of forgiveness (Heintzelman et al., 2014). Accordingly, the overall level of commitment may not be as explanatory of forgiveness as the level of commitment reported after the act of infidelity (Heintzelman et al., 2014). Future research could shed light on the association between commitment and forgiveness when faced with infidelity, as well as examine the role played by the different extradyadic behaviors in that relationship.

Ultimately, another variable that could influence our findings is accommodation. Moreover, given its close relationship with commitment (e.g., Rusbult et al., 1991, 1998), accommodation could present similar results in relation to forgiveness in light of infidelity. Through accommodation, people restrain their likelihood of engaging in destructive responses after a conflict with their partner. Furthermore, it is likely that people who show higher levels of commitment will accommodate themselves and use more constructive (rather than destructive) strategies when a conflict arises between both members of the relationship (e.g., Rusbult et al., 1991; Wieselquist et al., 1999). In this sense, the perception of a certain extradyadic behavior as indicative of infidelity could originate a conflict in the relationship—mainly in the offended person. Thus, people with high levels of commitment would show a greater willingness to adapt and use constructive strategies to face the problem with the transgressive partner and achieve a positive result for their relationship (e.g., discuss the problem with the partner and forgive him/her to restore the stability of the relationship). However, could this happen in the case of extradyadic behaviors of a sexual and technological nature? Furthermore, what if the commitment has been affected by such extradyadic behaviors? Further research is needed to address this complex relational process.

## Conclusion

In short, the studies described in this paper contribute to an improvement in the knowledge of the infidelity research field, showing that sexual and technological behaviors are considered more indicative of infidelity, and that technological infidelity can be as harmful as sexual infidelity, shedding light on the relevance of social networks and the Internet for the life of relationships. Likewise, the results provide evidence that unforgiveness—specifically motivation for revenge—can be considered by people with high anxious attachment to their partner to be an effective coping mechanism to counteract the negative affective state resulting from such betrayal. However, unforgiveness, in turn, is a significant source of stress and anxiety. In this regard, the results could also have implications for intervention because therapeutic practice focused on infidelity takes into consideration the option of forgiving as a means through which the physical and emotional well-being of the couple and of the person who suffers the betrayal can be restored, especially in people with anxious attachment to the partner, who may require more attention given the behavioral characteristics they exhibit in their relationships.

## Data Availability

The datasets generated for this study are available on request to the corresponding author.

## Ethics Statement

This research was carried out in accordance with the recommendations of Human Research Ethics Committee of the University of Granada. Participants provided informed consent in accordance with the 1964 Declaration of Helsinki.

## Author Contributions

AMB-M, IV-S, and FE designed the studies. ABM-M carried out the studies. AMB-M, IV-S, and FE analyzed and interpreted the data, and wrote the article.

## Conflict of Interest Statement

The authors declare that the research was conducted in the absence of any commercial or financial relationships that could be construed as a potential conflict of interest.

## Footnotes

1 Sex was analyzed as an exploratory variable between subjects to check if the results obtained differed according to the sex of the participants.

2 As in Study 1, sex was analyzed as an exploratory variable between-subjects to check if the results obtained differed according to the sex of the participants.

3 Given that sex did not show interaction effects in relation to anxious attachment and negative affect when the multiple regression analysis was performed, we proceeded to use Model 4 of simple mediation.

4 Different analyses of simple mediation were conducted according to the type of extradyadic behaviors and unforgiveness motivations. Regarding motivation for avoidance, no significant results were obtained for any of extradyadic behaviors. With respect to motivation for revenge, significant results were only obtained in relation to extradyadic Behavior of a sexual and technological nature.

5 Tables 5, 6 show the indirect effect of anxious attachment on the motivation for revenge through negative affect. Figure 1, 2 refer to the direct effect of anxious attachment on the motivation for revenge (c’).
